# Analysis of Potential Genes, Acute Phase Proteins and Hormonal Profiles Associated with Methicillin-Resistant *Staphylococcus aureus* (MRSA) Isolation from Pneumonic Sheep

**DOI:** 10.3390/vetsci12060584

**Published:** 2025-06-13

**Authors:** Hanan M. Alharbi, Eman A. Noaman, Ahmed El-Sayed, Mohamed T. Ragab, Amani Hafez, Attia Eissa, Ahmed Ateya, Khairiah M. Alwutayd, Manal A. Babaker, Asmaa Darwish

**Affiliations:** 1 Department of Biology, College of Science, Princess Nourah bint Abdulrahman University, P.O. Box 84428, Riyadh 11671, Saudi Arabia; hmalharbi@pnu.edu.sa (H.M.A.); kmalwateed@pnu.edu.sa (K.M.A.); 2 Department of Animal Health and Poultry, Animal and Poultry Production Division, Desert Research Center (DRC), Cairo 11753, Egypt; eman.noaman@drc.gov.eg (E.A.N.); decernes@drc.gov.eg (A.E.-S.); mohamedtaaat@drc.gov.eg (M.T.R.); amani.hafez@drc.gov.eg (A.H.); asmaa_vet25@drc.gov.eg (A.D.); 3 Department of Animal Medicine (Internal Medicine), Faculty of Veterinary Medicine, Arish University, Arish 45511, Egypt; attia.ahmed@vet.aru.edu.eg; 4 Department of Development of Animal Wealth, Faculty of Veterinary Medicine, Mansoura University, Mansoura 35516, Egypt; 5 Department of Chemistry, Faculty of Science, Majmaah University, Al Majmaah 11952, Saudi Arabia; m.babaker@mu.edu.sa

**Keywords:** MRSA *Staphylococcus aureus*, pneumonia, sheep, genetic polymorphism

## Abstract

*Staphylococcus aureus* is a significant bacterial pathogen responsible for a wide range of diseases in both humans and animals. This study aimed to investigate nucleotide sequence variations, gene expression patterns, and serum biomarkers, including acute phase proteins (APPs), hormonal fluctuations, and iron profile parameters in sheep affected by pneumonia. Additionally, the study focused on the isolation and characterization of *S. aureus* from pneumonic sheep, with particular emphasis on the prevalence of methicillin-resistant *S. aureus* (MRSA) strains. Blood samples were collected from both healthy and pneumonic sheep for gene expression and biochemical analyses, while nasal swabs from pneumonic sheep were used for bacterial isolation and identification. Out of 100 nasal swabs analyzed, 44% tested positive for *Staphylococcus* spp., and 61.4% of these were confirmed as *S. aureus* by PCR. The mecA gene, a key marker of methicillin resistance, was identified in 17 isolates (38.6% of the *S. aureus*-positive samples). MRSA isolates showed complete resistance to amoxicillin, cloxacillin, and erythromycin, and high resistance to penicillin, amoxicillin, and tetracycline; however, all MRSA strains remained fully susceptible to vancomycin. Gene expression analysis revealed that *TLR2*, *CLEC4E*, *PTX3*, *CXCL8*, and *IL15RA* were significantly upregulated (*p* < 0.05) in pneumonic ewes, while *SOCS3* expression was markedly downregulated. Sequence analysis of immune-related genes revealed notable nucleotide differences between healthy and affected animals. Furthermore, the pneumonic group exhibited significantly elevated levels of APPs, cortisol, and growth hormone, along with reduced levels of insulin, T3, and T4. These findings underscore the zoonotic risk posed by MRSA and emphasize the need for robust surveillance and antibiotic stewardship to control its spread. The study also highlights the importance of molecular diagnostics in accurately identifying MRSA and elucidating resistance mechanisms, thereby facilitating targeted treatment and informed management strategies.

## Simple Summary

*Staphylococcus aureus* is a significant bacterial pathogen responsible for a wide range of infections in both humans and animals. In this study, 100 pneumonic sheep and 100 clinically healthy sheep were examined to assess biochemical parameters and gene expression profiles. The findings revealed notable differences in gene expression, nucleotide sequences, and biochemical markers between healthy and pneumonic animals. Additionally, MRSA isolates displayed complete resistance to amoxicillin, cloxacillin, and erythromycin, along with high resistance to penicillin and tetracycline. However, all MRSA isolates remained fully susceptible to vancomycin. The observed alterations in molecular and biochemical marker profiles may contribute to the pathogenesis and clinical manifestations of pneumonia in sheep.

## 1. Introduction

Pneumonia is a major challenge in the global sheep industry, affecting approximately 30–40% of flocks and contributing to 40–70% of total sheep losses. In addition to mortality, pneumonia adversely impacts growth rates, leads to carcass condemnation, and substantially increases treatment and vaccination costs [1,2]. Collectively, these factors impose considerable financial burdens on sheep production systems.

Respiratory infections in sheep have a complex etiology involving interactions between the host immune response, environmental stressors, and a range of pathogenic organisms, including bacteria, mycoplasmas, viruses, and fungi. Major predisposing factors include transportation stress, overcrowding, abrupt weather changes, poor ventilation, and malnutrition. In Egypt, pneumonia is most prevalent during transitional seasons, particularly from autumn to early winter and from spring to early summer—when sheep experience sudden fluctuations between warm and cold temperatures. Additionally, elevated dust levels during spring and autumn can facilitate the deep penetration of pathogens into the respiratory tract [3,4,5,6,7].

*Staphylococcus aureus* is among the bacterial pathogens associated with pneumonia. This species is particularly significant due to its involvement in a wide range of diseases affecting both humans and animals. As reported in [8,9,10], *S. aureus* is a facultative anaerobic, Gram-positive, non-motile, and non-spore-forming coccus known for its remarkable adaptability and ability to persist in diverse environments. Approximately 25–40% of human and animal populations are asymptomatically colonized by *S. aureus*, primarily in the nasal passages and on the skin. In addition to causing cutaneous and soft tissue infections [11], *S. aureus* is a major contributor to foodborne illnesses, pneumonia, bacteremia, meningitis, sepsis, pericarditis, and other serious systemic infections [12,13].

The increasing antimicrobial resistance of *Staphylococcus aureus* infections is a significant public health concern. The emergence of methicillin-resistant *S. aureus* (MRSA) is attributed to bacterial strains that have developed resistance to methicillin and other β-lactam antibiotics. As reported in [14], these strains commonly exhibit resistance to a range of β-lactam antibiotics, including amoxicillin, penicillin, oxacillin, and methicillin. According to [15], monitoring the antibiotic resistance patterns of *S. aureus* isolates is crucial for minimizing treatment failures and implementing effective control measures.

Methicillin-resistant *Staphylococcus aureus* (MRSA) has emerged as a major pathogen in both human and veterinary medicine, with infections documented in hospital environments as well as within the community [16,17,18]. Since the first report of an MRSA strain in 1961 [19], its global prevalence has increased significantly, contributing to a rise in multidrug-resistant infections [20]. This rapid escalation in antibiotic resistance has posed serious challenges to conventional treatment strategies. MRSA, now considered one of the most common antimicrobial-resistant organisms worldwide, spreads efficiently in healthcare settings and exhibits high resistance to β-lactam antibiotics. This resistance is primarily mediated by the production of a modified penicillin-binding protein (PBP2a), which inhibits the action of β-lactam agents [21].

Initial treatment options for MRSA infections included daptomycin, linezolid, and vancomycin. However, MRSA has also developed resistance to these agents through various genetic adaptations, including alterations in *SCCmec* elements and single nucleotide polymorphisms (SNPs) within open reading frames (ORFs) [22]. The *mecA* gene, located on the staphylococcal cassette chromosome *mec* (*SCCmec*), encodes the penicillin-binding protein PBP2a and has undergone significant evolutionary modifications. These changes have played a critical role in the persistence and global dissemination of MRSA.

Compared to methicillin-sensitive *Staphylococcus aureus* (MSSA), MRSA infections are associated with poorer clinical outcomes [23,24]. The Centers for Disease Control and Prevention (CDC) and the World Health Organization (WHO) have identified MRSA as a significant global health threat [25]. The genetic diversity and antibiotic resistance patterns of *S. aureus* vary across geographical regions and host populations [26]. A major contributor to the emergence of MRSA in both humans and animals is the widespread and uncontrolled use of antibiotics [27]. Zoonotic transmission of MRSA has been extensively documented, with evidence supporting bidirectional transmission between humans and animals [28]. According to [29], asymptomatic colonization in the nasal and rectal regions further supports the role of animals as reservoirs for MRSA, facilitating its spread to humans and other animals.

The polymerase chain reaction (PCR) is considered one of the most rapid and effective techniques for detecting microbial infections in clinical samples. As noted in [30], PCR is particularly advantageous for identifying pathogens that are difficult to culture or require prolonged incubation periods. This method enables faster diagnosis and treatment, reduces the need for antimicrobial use, minimizes costs, and lowers the risk of adverse effects [31]. Although PCR-based assays enhance the molecular detection of MRSA, conventional bacterial culture remains the gold standard for definitive diagnosis [32].

Acute-phase proteins (APPs), primarily synthesized by the liver, are present in the blood of infected animals and play a crucial role in maintaining homeostasis, limiting tissue damage, and inhibiting microbial growth independently of antibodies [33]. During infection, inflammation, or other internal and external challenges, the concentration of APPs in plasma either increases (positive APPs) or decreases (negative APPs). These changes, which exhibit notable species-specific variation, provide valuable diagnostic and prognostic information in the context of infection and inflammation [34].

Advanced molecular genetic approaches have the potential to enhance animal health and support disease control efforts [35]. Several genetic markers, predominantly single nucleotide polymorphisms (SNPs), have been successfully identified to determine livestock susceptibility to various diseases [36,37,38]. This indicates that genetic variation among animals contributes to differences in disease vulnerability [39]. Marker-assisted selection (MAS) is a genomic strategy that uses these genetic markers as selection criteria, shifting the focus from observable disease symptoms to specific alleles at the DNA level. MAS improves selection accuracy and enables the identification of genetically resistant animals without the need for exposure to disease challenges, thereby offering a promising tool for breeding disease-resistant livestock [40].

Currently, limited information is available regarding the relationship between methicillin-resistant *Staphylococcus aureus* (MRSA) isolated from pneumonic sheep and changes in acute-phase proteins (APPs), hormonal levels, and gene expression. The development of effective preventive and therapeutic strategies is further hindered by the lack of reliable diagnostic tools and associated biomarkers for these conditions. This study aims to address these gaps by examining the gene expression patterns and serum profiles of APPs, hormonal markers, and iron-related parameters in Barki sheep infected with MRSA. Furthermore, the research will focus on the molecular identification of key antibiotic resistance genes, assess the prevalence of MRSA in nasal swabs, and characterize the antibiotic resistance profiles of the isolated strains. By elucidating the underlying molecular mechanisms, this approach seeks to provide essential insights to improve the management of ovine health and enhance disease monitoring strategies.

## 2. Material and Methods

### 2.1. Animals and Study Design

Two hundred Barki ewes weighing between 30 and 45 kg (mean ± SD: 37.5 ± 4.7) and between the ages of 3.5 and 5.5 (mean ± SD: 4.4 ± 0.6) were included in the study. The study was conducted between November 2023 and January 2024, corresponding to the late autumn and early winter seasons in the Matrouh Governorate, Egypt. During this period, the region experiences frequent fluctuations in temperature, increased humidity, and high atmospheric dust levels, particularly during early mornings and evenings. These environmental factors are well-documented contributors to respiratory stress and the onset of pneumonia in sheep. Recording these climatic conditions provides important context for interpreting the disease prevalence and biomarker fluctuations observed in the study. Following a comprehensive clinical examination in accordance with the technique described by [41], the ewes were split into two groups based on evaluations of their rumen, heart, and lung health, as well as other vital signs. The clinically healthy sheep in Group 1 (*n* = 100) were placed in the healthy control group (HCG), which had normal body temperature, pulse, respiration rate, clean eyes, no lacrimal or nasal discharge, normal posture, and appetite. One hundred sheep in the pneumonic group (PG) had clinical symptoms of respiratory disease, such as fever, belly breathing, mucopurulent nasal discharge, general weakness, appetite loss, and abnormal lung sounds (crackles and wheezes) identified through auscultation. The ewes were kept in semi-open, shaded quarters and fed 750 g of concentrated feed mixture (CFM) and 750 g of alfalfa hay per head that was given twice a day along with unlimited access to water. They also had access to green herbage, grass, berseem, darawa, and other natural grazing when it was available. The CFM composition consisted of 240 kg of wheat bran, 230 kg of soybean meal, 530 kg of maize, 5 kg of sodium chloride, 10 kg of calcium carbonate, 1 kg of premix, 0.5 kg of Netro-Nill, and 0.5 kg of Fylax.

### 2.2. Sampling

#### 2.2.1. Blood Sampling

Ten milliliters of fresh blood were drawn from each ewe in each group at 10 o’clock in the morning using a jugular vein puncture, and the blood was then split into three portions. The first and second parts were supplemented with EDTA and heparin calcium 5000 I.U., respectively, to disrupt the coagulation cascade. The EDTA blood was used for real-time PCR, and the second and third parts were centrifuged to extract serum and plasma, respectively, at 3000 r.p.m. for 20 min at 37 °C. They were then aliquoted and stored at −20 °C. The acute phase proteins (APPs), hormones, and iron profile parameters were then examined.

#### 2.2.2. Nasal Swabs

One hundred nose swabs were gathered in total. An assistant identified each animal and secured it while keeping it bound. A sterile cotton-tipped swab was placed within the nostril and rotated against the nasal cavity wall after the nasal exterior had been cleaned with 70% alcohol. Next, the swab was put into a sterile, labeled test tube with 10 mL of nutritional broth at 4 °C. Before being transported to the Microbiology Department laboratory at the Development of Matrouh Resources, Desert Research Center, Matrouh, the samples were kept in an icebox and kept under strict sanitary conditions for bacteriological examination.

### 2.3. Bacteriological Examination

#### 2.3.1. Isolation and Identification of *Staphylococcus aureus*

Each sample was inoculated into Baird-Parker agar plates supplemented with egg yolk tellurite using a 1 mL aliquot. Following a 24 to 48 h incubation period at 37 °C, suspicious colonies that were black, glossy, with a slight white border, and encircled by a clear zone that extended into the opaque medium began to expand.

The suspicious colonies were separated and streaked onto mannitol salt agar (MSA) medium, where they were incubated for 24 to 48 h at 37 °C to confirm the presence of *Staphylococcus aureus*. *S. aureus*-positive colonies were defined as those that showed yellow on MSA. Following the procedure outlined by [42], purified isolates were subjected to biochemical testing and Gram staining.

#### 2.3.2. Antimicrobial Susceptibility Testing

The disk diffusion approach was used to test for antibiotic susceptibility in accordance with the Clinical and Laboratory Standards Institute’s recommendations (CLSI, 2012). The following antibiotics were examined as follows: β-lactam antibiotics: methicillin (ME; 5 μg), ampicillin (A; 10 μg), cloxacillin (OB; 5 μg), oxacillin (OX; 1 μg), amoxicillin (AML; 25 μg), amoxicillin/clavulanic acid (AMC; 20 μg), penicillin (P; 10 IU), aminoglycosides, and gentamycin (G; 10 μg); and fluoroquinolones: ciprofloxacin (CIP; 5 μg). The approach followed the guidelines provided by Refs [43,44].

#### 2.3.3. Antimicrobial Susceptibility Testing (AST) for MRSA Strains

On Mueller–Hinton agar (Merck, Darmstadt, Germany), the traditional disk diffusion method was used to assess the susceptibility of positive *Staphylococcus aureus* isolates, including identified methicillin-resistant *S. aureus* (MRSA) strains, to fourteen antimicrobial drugs. Following the 0.5 McFarland standard (1.5 × 10^8^ CFU/mL), the test was conducted, and the Clinical and Laboratory Standards Institute (CLSI) criteria were followed for analysis [45]. The following fourteen antibacterial agents were tested as follows: β-lactam antibiotics: penicillin (P; 10 IU), amoxicillin (AML; 25 μg), amoxicillin/clavulanic acid (AMC; 20 μg), ceftriaxone (CTR), and cloxacillin (OB; 5 μg); aminoglycosides: gentamycin (G; 10 μg); fluoroquinolones: ciprofloxacin (CIP; 5 μg), levofloxacin (LEV), and ofloxacin (OFL); and other classes: vancomycin (VAN), cetirimoxazole (SMX), erythromycin (ERY), tetracycline (TET), and chloramphenicol (CHL). To establish susceptibility, the inhibition zones were measured in millimeters.

### 2.4. Molecular Confirmation of MRSA Strains and Detection of Virulence and Resistance Genes

In accordance with the manufacturer’s instructions, polymerase chain reaction (PCR) was used to molecularly confirm the presence of *Staphylococcus aureus* and methicillin-resistant *S. aureus* (MRSA). The positive control was *Staphylococcus aureus* subsp. aureus (ATCC^®^ 8096).

The QIAamp DNA Mini Kit (Qiagen GmbH, Hilden, Germany) was used to extract DNA from samples, with minor adjustments made to the manufacturer’s instructions. To summarize, 200 μL of the sample or tissue homogenate suspension was treated for 10 min at 56 °C with 10 μL of proteinase K and 200 μL of lysis buffer. Following incubation, the lysate was mixed with 200 μL of 100% ethanol. In accordance with the manufacturer’s recommendations, the sample was subsequently cleaned and centrifuged. A total of 100 μL of elution buffer was used to elute the nucleic acids.

Primers used in the study were supplied by Metabion (Planegg, Germany) and are listed in Table 1. The presence of the nuc gene was detected using specific primers, as described by [12]. Detection of the mecA gene, responsible for methicillin resistance, was carried out using primers reported by [46].

The following ingredients were used in the 25 μL reaction mixture used for PCR amplification: 1 μL of each primer (20 pmol concentration), 5.5 μL of nuclease-free water, 5 μL of DNA template, and 12.5 μL of EmeraldAmp Max PCR Master Mix (Takara, Kusatsu, Japan) were used. A 2720 thermal cycler from Applied Biosystems (Foster City, CA, USA) was used to carry out the procedures.

PCR products were electrophoresed using a voltage gradient of 5 V/cm on a 1.5% agarose gel (Applichem GmbH, Darmstadt, Germany) in 1× TBE buffer at room temperature. A total of 20 μL of uniplex PCR products and 40 μL of duplex PCR products were placed into each gel slot for the gel analysis. The fragment size was determined using the GeneRuler 100 bp Ladder (Fermentas, Darmstadt, Germany) and the GelPilot 100 bp Plus Ladder (Qiagen, Hilden, Germany). A gel documentation system (Alpha Innotech, San Leandro, CA, USA; Biometra, Gottingen, Germany) was used to display the gel, and computer software was used to evaluate the data.

### 2.5. Genetic Polymorphisms Between Healthy and Pneumonic Ewes

#### 2.5.1. Total RNA Extraction, Reverse Transcription and Quantitative Real Time PCR

In accordance with the manufacturer’s recommendations, total RNA was isolated from ewe blood using Trizol reagent (RNeasy Mini Kit, catalog no. 74104). The NanoDrop^®^ ND-1000 Spectrophotometer was used to quantify and qualify the amount of isolated RNA. Each sample’s cDNA was created in accordance with the manufacturing process (Thermo Fisher, Waltham, WA, USA, catalog no, EP0441). Using quantitative RT-PCR with SYBR Green PCR Master Mix (2× SensiFastTM SYBR, Bioline, CAT no: Bio-98002), the gene expression patterns for coding segments of genes encoding immunity (TLR2, CLEC4E, PTX3, SOCS3, CXCL8, and IL15RA) were evaluated. Via real-time PCR with the SYBR Green PCR Master Mix (Quantitect SYBR green PCR kit, catalog no. 204141), the relative mRNA level was measured.

Primers were created based on the *Ovis aries* sequence that was published in PubMed, as indicated in Table 2. As a constitutive control for normalization, the housekeeping gene GAPDH was employed. The reaction mixture, which had a total volume of 25 µL, contained 3 µL of total RNA, 4 µL of 5× Trans Amp buffer, 0.25 µL of reverse transcriptase, 0.5 µL of each primer, 12.5 µL of 2× Quantitect SYBR green PCR master mix, and 8.25 µL of RNase-free water. After putting the finished reaction mixture in a thermal cycler, the following procedure was run: reverse transcription for 30 min at 50 °C, primary denaturation for 10 min at 94 °C, 40 cycles of 94 °C for 15 s, annealing temperatures for 1 min as indicated in Table 2, and 72 °C for 30 s. A melting curve analysis was conducted at the conclusion of the amplification step to verify the PCR product’s specificity. Using the 2^−ΔΔCt^ technique, the relative expression of each gene per sample was compared to that of the GAPDH gene [47].

#### 2.5.2. DNA Sequencing and Polymorphism Detection

Removal of primer dimers, nonspecific bands, and other contaminants was performed prior to DNA sequencing. As described by [48], a PCR purification kit was used in accordance with the manufacturer’s instructions to purify real-time PCR products with the desired size (target bands) (Jena Bioscience # pp-201×s/ Jena, Germany). The Nanodrop (UV–Vis spectrophotometer Q5000/USA) was used to quantify the PCR product in order to produce high-quality results and guarantee sufficient concentrations and purity of the real-time PCR products [49]. PCR results containing the target band were sent for forward and reverse DNA sequencing using an ABI 3730XL DNA sequencer (Applied Biosystem, USA) in order to identify SNPs in genes examined in control and pneumonia-affected ewes. This was performed using an enzyme-chain terminator technique created by [50]. Chromas 1.45 and blast 2.0 tools were used to analyze the DNA sequencing data [51]. PCR results of the genes under investigation and reference sequences found in GenBank were compared, and differences were categorized as single-nucleotide polymorphisms (SNPs). Based on DNA sequencing data alignment, the MEGA6 software tool was used to compare the amino acid sequences of the genes under investigation among enrolled ewes [52].

### 2.6. Biochemical Analysis

IBL International Corp (Blainville, QC, Canada)^®^ ELISA kits were used to measure serum haptoglobin (Hp), plasma fibrinogen (Fb), and serum amyloid A (SAA). Turbidimetric analysis of serum transferrin (Tf) and caeruloplasmin (Cp) utilizing Elabscience USA^®^ (College Park, MD, USA) kits. Chemiluminescence immunoassay (CLIA) with Abnova^®^ kits (Taipei, Taiwan) for serum ferritin. Spectrophotometric measurement of serum iron (SI) and total iron-binding capacity (TIBC) using commercial kits from Biodiagnostic Company^®^ (Giza, Egypt). ELISA kits from MyBioSecure Company^®^ (Cheyenne, WY, USA) are used for hormonal assays (cortisol, insulin, TSH, T3, T4, and growth hormone (GH). Calculations were carried out as follows: Transferrin Saturation Percentage (Tf sat. %): Tf sat. % = SI/TIBC × 100, Unsaturated Iron-Binding Capacity (UIBC), and UIBC = TIBC − SI

### 2.7. Statistical Analysis

With SPSS software (version 23), an independent-sample T-test was used to do statistical comparisons between the investigated groups. The data observed in each group were tested for normality using the Kolmogorov–Smirnov test, and all parameters were not statistically significant, which indicates that the data are parametric (normally distributed), so the results were expressed as mean ± SD. Relationships between the genetic markers and serum profile of APP variables were evaluated using Pearson’s correlation. The difference in the frequencies of each gene SNP between pneumonic and healthy sheep was statistically assessed using the chi-square test to match the distribution of the recognized SNPs among the two groups using SPSS version 23, USA. A Linear Discriminant Analysis (LDA) was conducted to determine whether gene-level SNP averages could differentiate between pneumonic and healthy sheep. The 6 gene average scores served as predictor variables, and the health status (pneumonic vs. healthy) was the grouping variable. Statistical consequence was established at *p* < 0.05. A significance level of *p* < 0.05 was established. The sensitivity, specificity, cut-off values, and likelihood ratios (LR) for acute-phase proteins (APPs), hormonal and iron profiles were computed using GraphPad Prism software (version 8).

Predictive metrics and accuracy rates were determined using the following formulas:

PPV = true positive ÷ total positive × 100.

NPV = true negative ÷ total negative × 100.

Accuracy rate = (true positive + True negative) ÷ total population × 100.

Percentage of increase = (the mean value of the marker concentration in diseased group − the mean value of its concentration in CG) ÷ the mean value of its concentration in CG × 100.

## 3. Results

### 3.1. Clinical Findings

This study observed that respiratory distress symptoms such as rhinitis, congested mucous membranes, and a wet, harsh cough are common in affected sheep (Figure S1). The most frequent clinical signs included serous or mucoid nasal discharge, elevated respiratory and pulse rates, and increased rectal temperature exceeding 41 °C. Additional signs such as ataxia, ruminal atony, depression, and abnormal milk production were also recorded. Early-stage auscultation revealed pleuritic friction rubs, which progressively became muffled as the disease advanced.

### 3.2. Staphylococcus Isolates

The presence of *Staphylococcus aureus* and methicillin-resistant *S. aureus* (MRSA) was evaluated in 100 nasal swab samples. Initial identification of suspected *Staphylococcus* species was based on colony morphology on Baird-Parker agar, where colonies appeared black with a surrounding clear halo. Further confirmation was performed using mannitol salt agar (MSA), on which *S. aureus* colonies were characterized by a yellow coloration. Based on these culture characteristics, 44 out of the 100 samples (44%) were presumptively identified as *Staphylococcus* spp.

### 3.3. Molecular Identification

**Figure 1:**
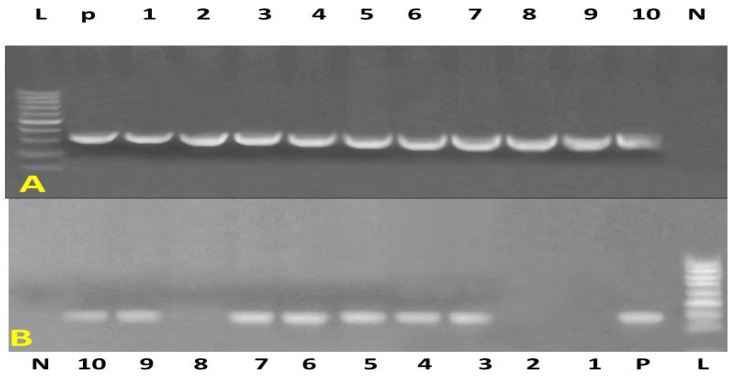
Agarose gel electrophoresis of PCR products. (**A**) Amplification of the nuc gene (395 bp). Lane 1: DNA ladder (100–1000 bp); Lane N: negative control; Lane P: positive control; Lanes 1–10: positive samples, except lanes 1, 7, and 10, which are negative. (**B**) Amplification of the mecA gene (310 bp). Lane 1: DNA ladder (100–1000 bp); Lane N: negative control; Lane P: positive control; Lanes 1–10: all positive samples.

PCR amplification of the nuc gene yielded a 395 bp amplicon, which was used for the molecular confirmation of *S. aureus* (Figure 1A). Further identification of MRSA isolates was achieved by detecting the mecA gene, characterized by a 310 bp amplicon (Figure 1B). Among the 44 suspected *Staphylococcus* isolates, 27 (61.4%) were confirmed as *S. aureus* based on nuc gene amplification. Of these, 17 isolates (38.6%) tested positive for the mecA gene and were classified as MRSA.

### 3.4. Antimicrobial Susceptibility of Staphylococcus aureus Isolated from Nasal Swabs of Sheep

The antibiotic susceptibility of *S. aureus* isolates obtained from nasal swabs was evaluated. As shown in Table 3, a high level of resistance was observed against cotrimoxazole and penicillin. Notably, all tested isolates exhibited multidrug resistance (MDR). The MRSA susceptibility profile revealed that 100% of isolates were resistant to amoxicillin, amoxicillin/clavulanate, cloxacillin, penicillin, erythromycin, chloramphenicol, tetracycline, and cotrimoxazole. Conversely, susceptibility was highest to levofloxacin (96%), followed by ofloxacin (70%) and gentamicin (15%). A detailed summary of these results is presented in Table 4.

### 3.5. Patterns for Transcript Levels of Immune Indicators

**Figure 2:**
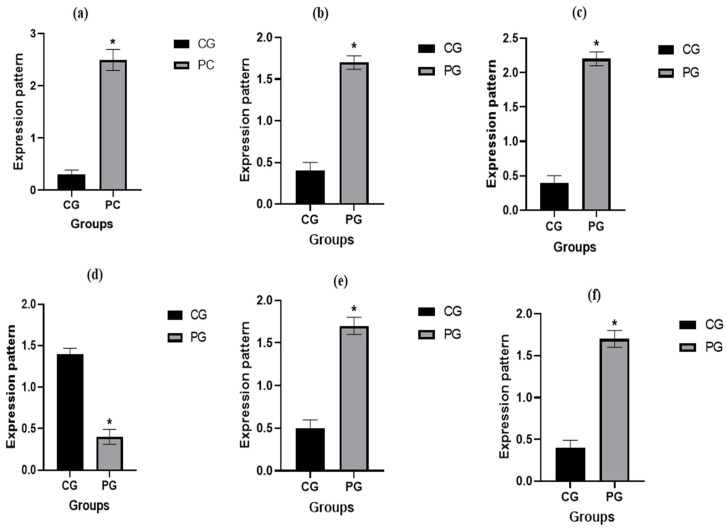
Normal and pneumonia-affected ewes have different immune genes: (**a**) = TLR2; (**b**) = CLEC4E; (**c**) = PTX3; (**d**) = SOCS3; (**e**) = CXCL8; and (**f**) = IL15RA transcript levels. When *p* is less than 0.05, significance is indicated by the symbol *. CG: control group; PG: pneumonic group.

Figure 2 illustrates the transcript patterns of the evaluated immunological markers. The genes *TLR2*, *CLEC4E*, *PTX3*, *CXCL8*, and *IL15RA* exhibited significantly higher expression levels in pneumonia-affected ewes compared to healthy controls. In contrast, *SOCS3* expression was significantly reduced in the affected group. Among pneumonic ewes, *TLR2* showed the highest mRNA expression level (2.52 ± 0.22), while *SOCS3* had the lowest (0.43 ± 0.16). Conversely, in healthy ewes, *SOCS3* demonstrated the highest mRNA level (1.40 ± 0.13), and *TLR2* had the lowest (0.37 ± 0.16).

### 3.6. Genetic Polymorphisms of Immune Genes

Using PCR-DNA sequence verdicts, the *TLR2* (354 bp), *CLEC4E* (443 bp), *PTX3* (363 bp), *CXCL8* (300 bp), *SOCS3* (480 bp) and *IL15RA* (378 bp) genes were found to have different SNPs in the amplified DNA bases linked to pneumonia. Based on the DNA sequence variations between immunological indicators examined in the ewes under study and the reference gene sequences retrieved from GenBank (Figures S1–S6), all of the identified SNPs were approved.

The coding DNA sequences of the affected ewes differed from those of the healthy ewes due to the exonic region abnormalities that were seen in Table 5 in all of the immunological markers tested. Using DNA sequencing of immune genes, seven SNPs were found; four of them were non-synonymous and three were synonymous. The four non-synonymous SNPs were associated with healthy ewes. The incidence of pneumonia, on the other hand, was linked to the identified three synonymous SNPs.

The *TLR2* (354 bp), *CLEC4E* (443 bp), and *IL15RA* (378 bp) genes each contain one recurrent SNP for each marker, according to DNA sequencing, which causes non-synonymous mutations L57E, C15Y, and S25N, respectively. The 300 bp *CXCL8* and 480 bp *SOCS3* genes both contained two synonymous SNPs. The amino acids 146T and 52L were linked to the two SNPs, G438C and C159T, respectively. Two recurrent SNPs were found when the *PTX3* (363 bp) gene was sequenced. In 30A, synonymous mutation G90C was implicated. In contrast, the C283T SNP caused the non-synonymous mutation L95F.

A significant difference was detected in the frequencies of all examined gene SNPs among pneumonic and healthy sheep (*p* < 0.005). Chi-square analysis was carried out for comparison of the distribution of all identified SNPs in all genes between pneumonic and healthy sheep. The total chi-square value showed significant variation among the identified SNPs in all genes between resistant and affected animals (*p* < 0.05) (Table 5).

Table 6 showed the discriminant analysis for the arrangement of kinds of genes and healthy status. The organizational consequences showed that the model properly classified 100% of the cases overall, either healthy sheep or pneumonic sheep. These results indicate that the SNP markers included in the model possess a good level of discriminatory power and may be useful as potential genetic indicators for pneumonia susceptibility in ewes.

### 3.7. Biochemical Profile

APPs (blood concentrations of acute-phase proteins (APPs) fibrinogen (Fb), haptoglobin (Hp), serum amyloid A (SAA), and ceruloplasmin (Cp) along with cortisol, growth hormone (GH), and thyroid-stimulating hormone (TSH) were significantly (*p* < 0.05) elevated in the pneumonia group (PG) compared to the control group (CG). In contrast, serum levels of insulin, triiodothyronine (T3), and thyroxine (T4) were significantly (*p* < 0.05) lower in PG. The iron profile of PG showed a significant (*p* < 0.05) increase in ferritin, total iron-binding capacity (TIBC), and unsaturated iron-binding capacity (UIBC), alongside a significant (*p* < 0.05) decrease in serum iron (SI), transferrin (Tf), and transferrin saturation percentage (Tf sat. %) compared to CG (Table 7).

Regarding the diagnostic and prognostic performance of APPs, hormones, and iron profile parameters, most markers demonstrated high sensitivity, specificity, positive predictive value (PPV), negative predictive value (NPV), and accuracy rates, all exceeding 70% at an area under the curve (AUC) > 0.7. An exception was TSH, which showed lower sensitivity and NPV (<70%). Likelihood ratios (LRs) varied among markers: SI, TIBC, and Tf sat. % exhibited low LRs (<5), T3 and GH had high LRs (~20), and the remaining markers showed moderate LRs (5–10). TSH and UIBC did not have calculable LRs due to their specificity values (LR = sensitivity/(1 − specificity)). Notably, among all predicted markers, Hp displayed the most pronounced percentage increase (Table 8).

### 3.8. Correlation Between Gene Expression Pattern of Immunological Markers and Serum Profile of Acute Phase Proteins in Healthy and Pneumonic Ewes

**Figure 3:**
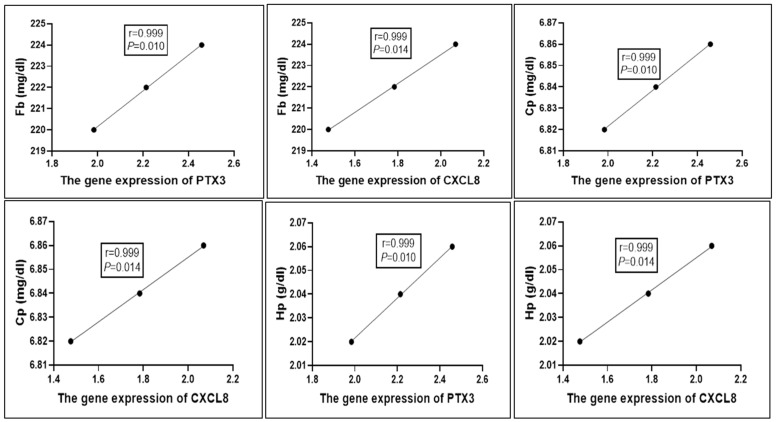
The correlation between the genetic markers and serum profile of acute phase proteins in CG and PG (Pearson’s Correlation test), considered significant when *p* < 0.05.

The correlation between the serum profile of acute phase proteins in the CG and PG and the gene expression patterns of immunological markers is shown in Figure 3. The mRNA levels of PTX3 and CXCL8 were positively correlated with the blood levels of Fb, Cp, and Hp (r = 0.999, *p* = 0.01).

## 4. Discussion

According to [53], pneumonia is a major cause of economic loss due to its high morbidity and mortality rates, frequent occurrence across sheep of all ages and breeds, and the associated impacts on growth rates and veterinary costs. Environmental stressors, such as extreme summer heat and harsh winter cold, increase the susceptibility of desert-dwelling animals to microbial infections, particularly respiratory diseases [54,55]. This study aims to investigate gene expression patterns and serum profiles of acute-phase proteins (APPs), hormonal markers, and iron profile parameters in Barki sheep infected with methicillin-resistant *Staphylococcus aureus* (MRSA). Additionally, it will determine the prevalence of MRSA in nasal swabs, analyze the antibiotic resistance profiles of isolated MRSA strains, and perform molecular identification of key antibiotic resistance genes. This comprehensive approach seeks to provide critical insights into the molecular mechanisms underlying these infections to support more effective management strategies for ovine health.

### 4.1. Clinical Examination

The pneumonic sheep in this study exhibited clinical signs including cyanotic mucous membranes, coughing, dyspnea, bilateral nasal discharge, elevated body temperature, and increased heart and respiratory rates compared to healthy controls. These findings align with observations reported in calves [56,57,58] and sheep [2,59,60]. The observed anorexia, depression, and lethargy may result from muscular weakness caused by intracellular potassium leakage, hyperkalemia, and hypoglycemia [61]. Additionally, infection and inflammation, along with the release of pyrogenic substances such as prostaglandins and stimulation of the hypothalamic thermoregulatory center, contribute to pain responses and hyperthermia [62].

### 4.2. Prevalence of Staphylococcus aureus in Pneumonic Sheep

*Staphylococcus aureus* is one of the most frequently isolated bacterial pathogens in pneumonic sheep. In this study, *S. aureus* was detected in 44% (44/100) of nasal swab samples. This prevalence is consistent with previous reports from Egypt, such as those by [63] (43%), [64] (42%), and [65] (40%). However, higher isolation rates have also been documented, including 50% [66], 55% [67], and 66% [68]. Conversely, lower recovery rates have been reported by [69] (15.5%), [70] (26%), and [71] (28.9%). The lowest recorded isolation rates are 1.23% in Southern Italy (Caruso et al., 2015) and 2.3% in Iran [72]. Management approaches, antibiotic use, isolation techniques, and environmental conditions can all have an impact on isolation frequencies [73].

### 4.3. Emergence of MRSA in Sheep

Due to its zoonotic potential, methicillin-resistant *Staphylococcus aureus* (MRSA) represents a significant public health concern, particularly among individuals in close contact with livestock [74]. The transmission of MRSA through the food chain further exacerbates this risk [75]. Timely and accurate diagnosis is essential for effective antibiotic therapy and infection control strategies [76]. Molecular techniques, including conventional PCR and real-time PCR, have substantially enhanced the detection of MRSA [77]. In the present study, the *mecA* gene—an indicator of methicillin resistance—was detected in 17 out of 44 (38.6%) *S. aureus* isolates, identifying them as MRSA. This prevalence is consistent with findings from Egypt [37] (36.6%), Nigeria [78,79] (34.7% and 43%, respectively), and Ethiopia [80] (32.4%). However, lower MRSA prevalence rates have been reported in Iran [81] (9.1%), Egypt [82] (25.3%), and European nations like France (6%), Ireland (5%), and the UK (2%) [83]. However, research in Egypt revealed rates as high as 85.7% [84] and even 100% [85], while [86] in Pakistan observed an extremely high MRSA incidence of 83%.

Several factors may account for the elevated MRSA frequency. Chief among them are inadequate infection control measures, including limited training opportunities, understaffing, heavy workloads, and resource constraints, which impede the implementation of standard precautions. In addition, widespread self-medication and the inappropriate use of antibiotics contribute significantly to resistance development. The increased detection of MRSA in Egyptian sheep may also be attributed to the expanded use of molecular diagnostic tools and the unregulated, extensive use of antibiotics in livestock production systems [87,88].

### 4.4. Antimicrobial Resistance of S. aureus and MRSA

Antimicrobial susceptibility testing showed that 21 out of 27 (77.7%) *S. aureus* isolates were susceptible to gentamicin, which is consistent with research by [89,90]. The 74% ofloxacin susceptibility was comparable to those from [91,92] in Nepal. Nonetheless, MRSA isolates showed high rates of resistance: 100% susceptibility to vancomycin, 96% susceptibility to levofloxacin, 70% susceptibility to ofloxacin, and 100% resistance to amoxicillin, amoxicillin/clavulanate, cloxacillin, penicillin, erythromycin, chloramphenicol, tetracycline, and cotrimoxazole. According to [93], the mecA gene, which encodes penicillin-binding protein 2a (PBP2a), is largely responsible for MRSA’s resistance mechanism. This gene allows the bacteria to withstand β-lactam drugs. As a result of its high prevalence in Europe, North Africa, the Middle East, and East Asia, MRSA has now gained international attention [27]. Antibiotic resistance in *S. aureus* has been greatly exacerbated by the careless use of antibiotics without a prescription in underdeveloped nations [89]. According to investigations conducted in Owerri, Nigeria [94], cloxacillin was previously very successful against *S. aureus*, with 78% susceptibility and 85.4% susceptibility, respectively. But according to recent research, such as [95], there has been a notable drop in susceptibility to 38.5%, most likely as a result of the increase in methicillin resistance. Likewise, this study’s findings on tetracycline, chloramphenicol, penicillin, and cotrimoxazole resistance are consistent with those from Eritrea [96] and Nigeria [89,95].

### 4.5. Nucleotide Sequence Variants of Investigated Immune Genes Linked to Pneumonia

We investigated the alterations in the immunological states of ewes with pneumonia in comparison to healthy ewes by assessing the mRNA levels of immune genes. The expression of the genes *TLR2*, *CLEC4E*, *PTX3*, *CXCL8*, and *IL15RA* was much higher in the afflicted ewes than in the healthy ones. However, SOCS3 levels were decreased. Defensin, *SP110*, *SPP1*, *BP1*, *A2M*, *ADORA3*, *CARD15*, *IRF3*, and *SCART1* are among the immune genes whose levels differ in goats with and without pneumonia [97]. Gene expression profiling has connected viral pneumonia in sheep to complement genes and Toll-like receptors (TLRs) [98].

This study used a PCR-DNA sequencing technique to characterize the immunological (*TLR2*, *CLEC4E*, *PTX3*, *CXCL8*, *SOCS3* and *IL15RA*) genes in ewes with pneumonia and healthy ewes. The results demonstrate that there are differences in the SNPs involving the two categories. It is crucial to stress that, in contrast to the similar datasets obtained from GenBank, the polymorphisms discovered and made available in this context offer extra information for the evaluated indicators. This link is initially shown by the *Ovis aris* gene sequences used in our study, which were published in PubMed.

Susceptibility to pneumonia in ruminants has been investigated using the candidate gene approach, revealing notable differences in the nucleotide sequences of immune-related genes between pneumonic and healthy lambs [99]. In pneumonia-affected sheep, the expression levels of genes such as *IL-1α*, *IL1B*, *IL6*, *TNF-α*, *LFA-1*, *CR2*, *IL17*, *IL13*, *DEFB123*, *SCART1*, *ICAM1*, *NOS*, and *HMOX1* were significantly upregulated compared to resistant ewes. Conversely, genes involved in anti-inflammatory and antioxidant responses (*IL10*, *SOD1*, *CAT*, *GPX1*, and *NQO1*) were downregulated. For example, susceptibility or resistance to *Mycoplasma ovipneumoniae* in sheep has been associated with polymorphisms in the *MHC-DRB1* exon 2 region [100]. The influence of *TMEM154* gene variations on sheep susceptibility to ovine progressive pneumonia virus following natural exposure was examined by [101]. Additionally, single nucleotide polymorphisms (SNPs) linked to pneumonia susceptibility and resistance were identified in several immune-related genes—SLC11A1, CD14, CCL2, TLR1, TLR7, TLR8, TLR9, defensin, SP110, SPP1, BP1, A2M, ADORA3, CARD15, IRF3, and SCART1—in Baladi goats affected by pneumonia, compared to a healthy control group, using PCR-DNA sequencing [97].

We found that the *TLR2* (354 bp), *CLEC4E* (443 bp), and *IL15RA* (378 bp) genes each had one recurrent SNP for each marker, which leads to non-synonymous mutations L57E, C15Y, and S25N, respectively, based on DNA sequencing. Goats, cattle, and buffalo preserve the modified base in the TLR2 gene (GenBank accession numbers XM_005217446.5, JN608796.1, and JQ342089.1, respectively). A conserved G43A SNP for the CLEC4E gene is found in bighorn sheep (*Ovis canadensis*) (GenBank accession number XM_069585824.1). Additionally, scimitar-horned oryx (Oryx dammah; GenBank accession code XM_040248264.1) databases contain the A74G SNP for the *IL15RA* gene.

There were two synonymous SNPs in the 300 bp *CXCL8* and 480 bp *SOCS3* genes. The amino acids 146T and 52L, respectively, were linked to the two SNPs, G438C and C159T. It was found that the altered nucleotide in SOCS3 was conserved when compared to the genomes of cattle, buffalo, and bighorn sheep (GenBank accession numbers NM_174466.2, XM_025279609.3, and ON390895.1, respectively). Similarly, databases for cattle, buffalo, and bighorn sheep were found to include the C159T SNP in *CXCL8*, which has the GenBank accession numbers NM_173925.2, FJ595833.1, and EF524263.1, respectively.

Sequencing of the *PTX3* (363 bp) gene revealed two recurrent SNPs. G90C was identified as a synonymous mutation in 30A. Conversely, the non-synonymous mutation L95F was brought on by the C283T SNP. The cattle has a conserved G90C SNP (GenBank accession number NM_001076259.2), and the goat has a conserved C283T mutated base (GenBank accession number XM_018048475.1).

Immune gene DNA sequencing revealed seven SNPs, three of which were synonymous and four of which were non-synonymous. A correlation was found between the four non-synonymous SNPs and healthy ewes. In contrast, three synonymous SNPs were shown to be associated with the incidence of pneumonia. Mutation is the central cause of selection and adaptability [102]. Exonic area mutations were presented in all examined markers under investigation in this case, leading to different coding DNA sequences in pneumonic as opposed to healthy ewes. Four non-synonymous and three synonymous SNPs were identified through DNA sequencing of investigated genes. Protein sequences are altered by non-synonymous mutations, and natural selection usually targets animals with these changes [102]. Genetic discrepancy caused by non-synonymous SNPs adjusts the fixed amino acid at the mutant site, potentially leading to structural and practical deviations in the mutated protein [103]. It was long thought that there was either no selection on synonymous mutations or very little selection [102]. The examined genes must be precisely characterized at the molecular level in order to comprehend the physiological alterations between pneumonic and healthy ewes in terms of resistance and susceptibility. According to our research, polymorphisms based on the translated DNA sequence of ewes are more valued than intronic regions.

According to the afoementioned findings, the C169T (L→F) substitution in TLR2 and G43A (C→Y) in CLEC4E were predicted to be “probably damaging” due to alterations in conserved hydrophobic domains critical for receptor-ligand binding. The A74G (S→N) variant in IL15RA lies within a domain involved in cytokine receptor activity and was also predicted to have a moderate impact on protein function [102,103].

In the face of microbes, the first line of defense depends on innate immune systems, especially Toll-like receptors and antimicrobial peptides [104]. Pathogen-associated molecular forms are seen in the ten Toll-like receptor (TLR) family members that are typically encoded in the mammalian genome [105]. TLR2 is able to identify bacterial lipids like lipoteichoic acid (LTA) [54].

Different cell types express the glycoprotein pentraxin 3 (PTX3) in response to primary inflammatory stimuli, including those mediated by TNFα, IL-1β, and TLR agonists [106,107]. According to [108], the PTX3 gene is primarily responsible for regulating innate resistance to pathogens and inflammatory responses. It also functions as an antibacterial agent that may help protect the mammary gland against chronic and subclinical infections. According to several studies, the goats’ udder’s first line of defense against *S. aureus* infection is PTX3, which is markedly up-regulated [108,109]. In the examined Holstein and Montbéliarde dairy cows, single nucleotide polymorphisms (SNPs) and gene expression profile changes related to mastitis resistance/susceptibility were found in the PTX3 gene [110].

As an immune response gene linked to genetic resistance and susceptibility to a wide range of diseases, CLEC4E is expressed on the surface of macrophages [111] and is crucial in the immune system’s recognition of bacterial glycolipids [112]. In goats vulnerable to nematode infection, Ref. [113] demonstrated that CLEC4E was a down-regulated gene implicated in the immune response system. When examining the in vivo transcriptional response of mammary epithelial cells during the early phases of infection with *Staphylococcus aureus*, CLEC4E was elevated in other goat investigations [112].

Numerous cell types, including macrophages, epithelial cells, and endothelial cells, produce the chemokine interleukin-8 (IL8), also known as chemokine (C-X-C motif) ligand 8, or CXCL8 [114]. This chemokine acts on CXCR1 and CXCR2 receptors and is one of the main mediators of the inflammatory response [115].

A cytokine receptor with a high affinity for interleukin 15 (IL15) is encoded by the IL15RA gene. The IL-15 signaling pathways and their main biological effects on various immune cell types and innate immunity systems are examples of similar pathways. SNPs in IL15RA were linked to macro-pathogen richness in humans [116]. Goats’ resistance to gastrointestinal illnesses and mastitis was found to be influenced by the IL15RA gene [117].

A protein that inhibits cytokine signaling, SOCS3 belongs to the suppressors of cytokine signaling (SOCS) family [118]. The role of mammary epithelial cells in the immune response during the initial phases of a bacterial infection with *S. aureus* in goats was investigated by [112].

The entire collection of genes in the genome that are consistently and effectively expressed under a range of physiological and pathological circumstances is referred to as the “transcriptome” [119]. It has proven useful in identifying new therapeutic or diagnostic targets and is widely employed in inflammatory illnesses to assess the immune system and detect pathogenic, diagnostic, and prognostic markers [120].

This alteration in immunological marker expression patterns in ewes with pneumonia may be due to the fact that wounded tissues are more susceptible to free radical reactions than healthy tissues [121]. By binding to cell surface receptors, pneumonia also produces a range of cellular immunological components that control and regulate the immune system and inflammatory response [122]. The intricate interactions among these immunological components have been demonstrated by numerous studies. In the case of acute-phase reactive proteins, complement proteins, and other immune globulins, for instance, the interaction of these proteins results in a complex network structure [123]. Therefore, our hypothesis is that infectious pneumonia may have affected most of the sheep in our study. Furthermore, our real-time PCR data clearly shows that sheep with pneumonia had a marked inflammatory response.

### 4.6. Biochemical Markers

APP levels in the PG were considerably higher than those in the CG, which is indicative of the biochemical changes in the pneumonic sheep in this investigation. This outcome was consistent with earlier research showing elevated APP concentrations in pneumonia cases in a variety of animal species [4,124,125]. In the innate immune response, APPs are essential because they serve as the body’s initial line of defense against infection, damage, and cancer. When disease strikes, APPs, which are produced by hepatocytes in response to pro-inflammatory cytokines, quickly increase in number and stay in the bloodstream for a long time. While each protein has distinct qualities, APPs work in concert to identify infections and limit microbial proliferation. Through the binding of free hemoglobin, Hp reduces oxidative damage and denies bacteria the iron they need to proliferate. This has both bacteriostatic and antioxidant benefits. Because it remains in the bloodstream longer than SAA, Hp is also a good predictor of tissue damage and disease consequences. SAA, on the other hand, reacts quickly to infections by binding bacteria and making it easier for neutrophils and macrophages to absorb them [1,4,124,125]. Because of its anti-inflammatory and antioxidant qualities, Cp helps to regulate the immune system. As an extracellular peroxide scavenger, it transforms poisonous ferrous iron into its harmless ferric form, neutralizes dangerous reactive oxygen species, and decreases neutrophil adherence to the endothelium. According to [4,124,125], Fb is essential for regulating hemostasis, coordinating the inflammatory response, and promoting tissue healing. When taken as a whole, these APPs strengthen the body’s defenses against infections, inflammation, and the initiation of healing processes.

Pneumonia causes major hormonal changes in addition to being an inflammatory illness. The complex interaction between the immunological and endocrine systems is highlighted by this study, which shows that PG patients had significant hypercortisolemia, hypoinsulinemia, increased growth hormone (GH), and decreased T3 and T4 levels. The endocrine system tries to maintain immune function by reversing the significant metabolic changes brought on by the immunological response during infection [126]. The endocrine system tries to maintain immune function by reversing the significant metabolic changes brought on by the immunological response during infection [126]. The hypothalamic–pituitary–adrenal (HPA) axis is directly stimulated by pro-inflammatory cytokines, which results in the release of adrenocorticotropic hormone (ACTH) and adrenal activation, which raises cortisol levels. The increase in cortisol is necessary for preventing inflammation-induced hypoglycemia and providing a sufficient amount of energy for vital physiological functions as well as immunological defense systems. Increased pituitary GH production, decreased cellular glucose absorption, and impaired insulin synthesis are just some of the metabolic consequences of hypercortisolemia [127,128]. It also supports energy homeostasis by suppressing thyroid function and encouraging gluconeogenesis and glycogenolysis. A powerful immunomodulator, cortisol inhibits the generation of pro-inflammatory cytokines and shifts immunological activity from innate to humoral immunity, in addition to its metabolic function [126].

Thyroid hormone (T3 and T4) reduction, increased growth hormone, and hypoinsulinemia in PG are all consistent with these adaptive endocrine responses. In order to restore thyroid hormone levels, a corresponding increase in TSH most likely serves as a compensatory mechanism. Because of this complex hormonal interaction, the endocrine system plays a vital role in preserving immunological and metabolic homeostasis during pneumonia [126].

PG’s iron profile showed hyperferritinemia, hypoferremia, and hypotransferrinemia. These alterations were influenced by the immunological response in multiple ways. To prevent the invasive bacteria from growing and multiplying, the activated pro-inflammatory cytokines obstruct their access to iron. Therefore, they decrease intestinal iron absorption, decrease transferrin activity, promote hepcidin release (which competes with transferrin for iron), and promote iron storage as ferritin [3,129]. Because ferritin is a positive acute phase reactant and transferrin is a negative acute phase reactant, the acute phase response, which typically accompanies respiratory and lung diseases, also contributes to these changes [130,131]. Furthermore, the observed hyperferritinemia may be a result of oxidative stress associated with pneumonia, as the accumulated free radicals attack and break the membrane of the red blood cells, resulting in the release of a significant amount of free iron. Consequently, the body increased ferritin production to eliminate these detrimental consequences of free iron [129]. This hypoferremia is rationally explained by the increased TIBC and UIBC and decreased Tf sat. %.

APPs, hormones, and iron profile measures showed high sensitivity, specificity, PPV, NPV, and accuracy rate (except for TSH), with an AUC > 0.7, in terms of their diagnostic and prognostic value. These results are somewhat consistent with earlier studies that used APPs for reliable indicators of pneumonia in sheep and other animals. The study’s potential use of iron profile and hormonal parameters as useful disease indicators, however, is a significant new finding [4,124,125]. The study strongly suggests UIBC because of its exceptional diagnostic performance, T3 and GH because of their high LRs, and Hp because of its distinct percentage increase among the tested markers. These results highlight the potential benefits of combining iron profile measures, hormone markers, and APPs for improved pneumonia diagnosis and prognosis.

The current work has several restrictions that should be addressed in upcoming research. First, the study was conducted on a small number of sheep; therefore, studies involving larger sample sizes are needed. Second, the study focused on a single breed, so applying this research to different calf breeds would provide more accurate health assessments. Third, only a limited set of immune genes were examined, indicating the need for broader gene profiling in subsequent studies. Fourth, pharmacokinetic/pharmacodynamic (PK/PD) studies, therapeutic efficacy trials, and field validation across multiple regions must be integrated for a more thorough and representative treatment approach. As a result, studies that expand the geographical scope and include clinical treatment trials are necessary to verify the effectiveness, safety, and economic viability of the recommended therapeutic agents. This would ensure that the selected treatments are not only microbiologically effective but also clinically practical and sustainable under diverse field conditions.

## 5. Conclusions

This study highlights the significant prevalence of methicillin-resistant *Staphylococcus aureus* (MRSA) among pneumonic sheep in Egypt and underscores its multidrug-resistant profile, particularly its complete resistance to several β-lactam antibiotics. Gene expression analysis revealed significant upregulation of immune-related markers (TLR2, CLEC4E, PTX3, CXCL8, IL15RA) and notable SNP variations associated with pneumonia susceptibility. Biochemical profiling showed marked alterations in acute-phase proteins, hormones, and iron metabolism, supporting their potential diagnostic and prognostic value. These findings suggest that integrating molecular diagnostics, SNP-based genetic markers, and serum biomarker profiling can enhance early detection, targeted therapy, and selective breeding strategies to mitigate pneumonia in sheep flocks.

## Figures and Tables

**Table 1:** Primers sequences, target genes, amplicon sizes and cycling conditions for MRSA and some virulence genes.

Target Gene	Primers Sequences	Amplified Segment (bp)	Primary Denaturation	Amplification (35 Cycles)			Final Extension	Reference
				Secondary Denaturation	Annealing	Extension		
Staph *nuc*	ATATGTATGGCAATCGTTTCAAT	395	94 °C 5 min.	94 °C 30 sec.	55 °C 40 sec	72 °C 40 sec.	72 °C 10 min.	[12]
	GTAAATGCACTTGCTTCAGGAC							
Staph *mecA*	GTA GAA ATG ACT GAA CGT CCG ATA A	310	94 °C 5 min.	94 °C 30 sec.	50 °C 30 sec	72 °C 30 sec	72 °C 10 min	[46]
	CCA ATT CCA CAT TGT TTC GGT CTA A							

**Table 2:** Forward-reverse oligonucleotide-based real-time PCR primers for the immunological genes being studied.

Investigated Marker	Primer	Product Size (bp)	Annealing Temperature (°C)	GenBank Isolate	Origin
*TLR2*	F5′-GCCTGGCTCCAGGCCAAGAGGA-3 R5′-TCCTCTTGGCCTGGAGCCAGGC-3′	354	58	NM_001048231.1	
*CLEC4E*	F5′-ATTCATCCACATCACCAGCATCA-3 R5′-GTGACCCTCGACCACCTGGTC-3′	443	55	XM_042247586.1	Present Research
*PTX3*	F5′-CTGGAGGAGCTGCGGCGGACGC-3′ R5′-GGTGCTGCACAGATGGGTCCATG-3′	363	58	XM_004003220.5	
*SOCS3*	F5′-ACCTTCCTCATCCGCGACAGCTC-3′ R5′-ATCGTACTGGTCCAGGAACTC-3′	480	58	XM_027974200.3	
*CXCL8*	F5′-ATGACTTCCAAGCTGGCTGTTG-3′ R5′-ATCTTGCTTCTCAGCTCTCTTC-3′	300	55	NM_001009401.2	
*IL15RA*	F5′-CCGGCCACGCCGGGCATCACCTG-3′ R5′-CACCAGGCACACTGCAAAGAC-3′	378	58	XM_042230436.2	
*GAPDH*	F5′-GTGAAGGTCGGAGTGAACGG-3′ R5′-TTGACTGTGCCGTGGAACTT-3′	173	58	HM043737.1	

**Table 3:** Antibiotic sensitivity and resistance pattern of *Staphylococcus aureus* from isolates.

Type of Antibiotic	No. Sensitive/27	No. Resistant/27	% Sensitive	% Resistant
Gentamicin	21	6	77.7	22.2
Amoxycillin	8	19	29.6	70.4
Amoxycillin/clavullanate	17	10	63	37.0
Ceftriaxone	20	7	74	26
Cloxacillin	10	17	37	63
Ciprofloxacin	21	6	77.7	22.2
Levofloxacin	27	0	100	0
Penicillin	1	26	3.7	96.3
Erythromycin	14	13	51,8	48
Chloramphenicol	16	11	59.2	40.7
Ofloxacin	20	7	74	26
Tetracycline	8	19	29.6	70.3
Cotrimoxazole	4	23	14.8	85.2
vancomycin	17	0	100	0

**Table 4:** Antibiotic sensitivity pattern of methicillin resistant isolates.

Type of Antibiotic	No. Sensitive/17	No. Resistant/17	% Sensitive	% Resistant
Gentamicin	2	15	11.7	88.3
Amoxycillin	0	17	0	100
Amoxycillin/clavullanate	0	17	0	100
Ceftriaxone	7	10	41.2	58.8
Cloxacillin	0	17	0	100
Ciprofloxacin	9	8	53	47
Levofloxacin	16	1	94	5.8
Penicillin	0	17	0	100
Erythromycin	0	17	0	100
Chloramphenicol	0	17	0	100
Ofloxacin	12	5	70.6	29.4
Tetracycline	0	17	0	100
Cotrimoxazole	0	17	0	100
vancomycin	17	0	100	0

**Table 5:** Pneumonic and healthy ewes’ immunological marker distributions with a single base differential and possible genetic change.

Gene	SNPs	Healthy *n* = 100	Pneumonia *n* = 100	Total *n* = 200	Chi Square X^2^	*p* Value	Kind of Inherited Change	Amino Acid Order and Sort
*TLR2*	C169T	73/100	-/100	73/200	115	<0.005	Non-synonymous	57 L to F
*CLEC4E*	G43A	59/100	-/100	59/200	83.69	<0.005	Non-synonymous	15 C to Y
*PTX3*	G90C	-/100	84/100	84/200	144.8	<0.005	Synonymous	30 A
	C2383T	39/100	-/100	39/200	48.45	<0.005	Non-synonymous	95 L to F
*SOCS3*	G438C	-/100	48/100	48/200	63.13	<0.005	Synonymous	146 T
*CXCL8*	C154T	-/100	74/100	74/200	117.5	<0.005	Synonymous	52 L
*IL15RA*	A74G	43/100	-/100	43/200	54.8	<0.005	Non-synonymous	25 S to N

**Table 6:** Discriminant analysis for classification of type of genes and healthy status of examined sheep.

		Predicted Group Membership		Total
		Healthy	Diseases	
Count	Healthy	100	0	100
	Diseased	0	100	100
%	Healthy	100.0	0.0	100.0
	Diseased	0.0	100.0	100.0

**Table 7:** Biochemical parameters of control and pneumonic groups.

Parameters	Control Group	Pneumonic Group
Fb (mg/dL)	122.01 ± 8.49	225.01 ± 3.47 *
Cp (mg/dL)	2.30 ± 1.15	6.24 ± 0.02 *
Hp (g/dL)	0.15 ± 0.02	2.59 ± 0.49 *
SAA (mg/L)	2.32 ± 0.15	6.96 ± 0.16 *
Cortisol (μg/dL)	1.79 ± 0.16	6.44 ± 0.05 *
Insulin (μIU/mL)	8.41 ± 0.15	7.08 ± 0.17 *
T3(ng/mL)	1.74 ± 0.15	1.02 ± 0.02 *
T4 (μg/mL)	0.85 ± 0.08	0.65 ± 0.02 *
TSH (μIU/mL)	0.010 ± 0.002	0.022 ± 0.012 *
GH (ng/dL)	12.39 ± 1.47	16.80 ± 0.05 *
SI (μg/dL)	106.89 ± 2.46	89.13 ± 1.62 *
TIBC (μg/dL)	327.39 ± 2.16	341.42 ± 3.11 *
UIBC (μg/dL)	220.50 ± 2.24	252.29 ± 3.72 *
Transferrin(mg/dL)	124.65 ± 2.74	86.47 ± 0.22 *
Tf sat. %	32.65 ± 0.66	26.11 ± 0.56 *
Ferritin (ng/mL)	13.60 ± 1.05	19.35 ± 0.53 *

Significant differences between the two groups were indicated by (*) when *p* < 0.05.

**Table 8:** Roc curve analysis of APPs, hormones, and iron profile parameters in PG compared to CG.

Parameters	Cut-Off Point	Sensitivity	Specificity	LR	PPV	NPV	AR	% of Increase or Decrease
Fb (mg/dL)	132.50	100%	85%	6.67	90.91%	100%	94%	84.41%
Cp (mg/dL)	3.60	100%	80%	5	88.24%	100%	92%	171.30%
Hp (g/dL)	0.185	100%	90%	10	93.75%	100%	96%	1626.67%
SAA (mg/L)	2.45	100%	80%	5	88.24%	100%	92%	200%
Cortisol (μg/dL)	1.99	100%	85%	6.67	90.91%	100%	94%	259.77%
Insulin (μIU/mL)	8. 26	100%	80%	5	88.24%	100%	92%	−15.81%
T3(ng/mL)	1.57	100%	95%	20	96.77%	100%	98%	−41.37%
T4 (μg/mL)	0.78	100%	80%	5	88.24%	100%	92%	−23.52%
TSH (μIU/mL)	0.015	60%	100%	-	100%	62.50%	76%	120%
GH (ng/dL)	14.39	100%	95%	20	96.77%	100%	98%	35.59%
SI (μg/dL)	106	100%	75%	4	85.71%	100%	90%	−16.61%
TIBC (μg/dL)	328	100%	75%	4	85.71%	100%	90%	4.28%
UIBC (μg/dL)	232.9	100%	100%	-	100%	100%	100%	14.41%
Transferrin(mg/dL)	121.50	100%	85%	6.67	90.91%	100%	94%	−30.62%
Tf sat. %	32.51	100%	75%	4	85.71%	100%	90%	−20.03%
Ferritin (ng/mL)	14.50	100%	80%	5	88.24%	100%	92%	42.28%
LR = 0.5–5: low; LR = 5–10: moderate; LR > 10: high.								

## Data Availability

Upon reasonable request, the appropriate author will share supporting data for the study’s conclusions.

## Supplementary Materials

The following supporting information can be downloaded at https://www.mdpi.com/article/10.3390/vetsci12060584/s1, Figure S1: Sheep showing signs of pneumonia. Figure S2: Representative alignment of the TLR2 gene (354 bp) between healthy (H) and pneumonia-affected ewes (P) compared to reference GenBank accession number (NM_001048231.1). Figure S3: Representative alignment of the CLEC4E gene (443 bp) between healthy (H) and pneumonia-affected ewes (P) compared to reference GenBank accession number (XM_042247586.1). Figure S4: Representative alignment of the PTX3 gene (363 bp) between healthy (H) and pneumonia-affected ewes (P) compared to reference GenBank accession number (XM_004003220.5). Figure S5: Representative alignment of the SOCS3 gene (480 bp) between healthy (H) and pneumonia-affected ewes (P) compared to reference GenBank accession number (XM_027974200.3). Figure S6: Representative alignment of the CXCL8 gene (300 bp) between healthy (H) and pneumonia-affected ewes (P) compared to reference GenBank accession number (NM_001009401.2). Figure S7: Representative alignment of the IL15RA gene (378 bp) between healthy (H) and pneumonia-affected ewes (P) compared to reference GenBank accession number (XM_042230436.2).
