# Supplementary material for: Analysis of Potential Genes, Acute Phase Proteins and Hormonal Profiles Associated with Methicillin-Resistant Staphylococcus aureus (MRSA) Isolation from Pneumonic Sheep
Source: Vet Sci. 2025 Jun 13;12(6):584. doi: 10.3390/vetsci12060584 (PMC12197726; doi:10.3390/vetsci12060584)
Supplement: Supplementary file 1 [file vetsci-12-00584-s001.zip › vetsci-3681271-supplementary.pdf]

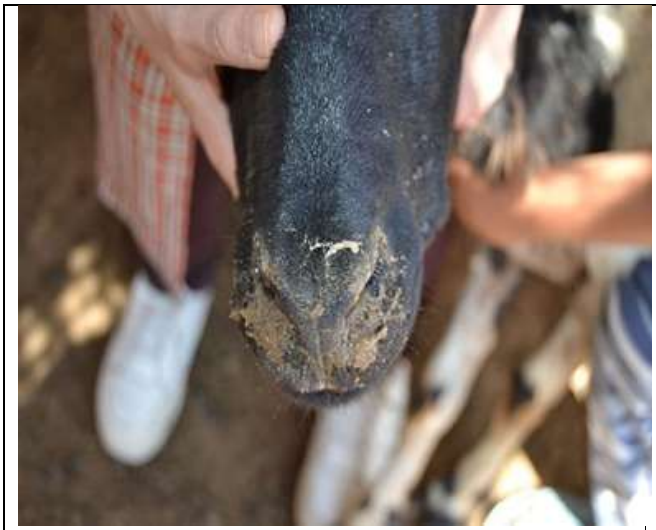
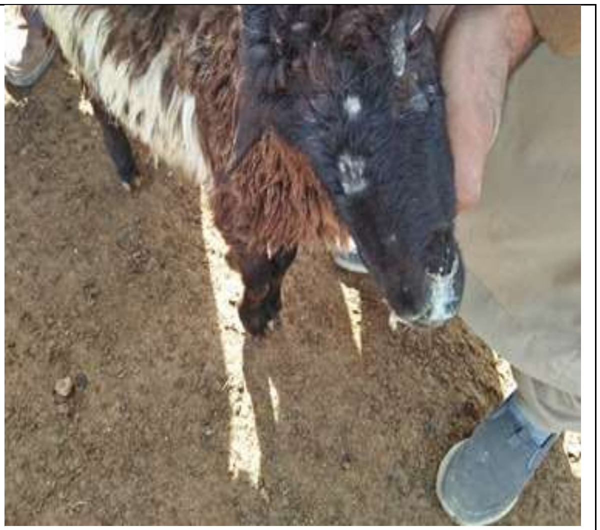
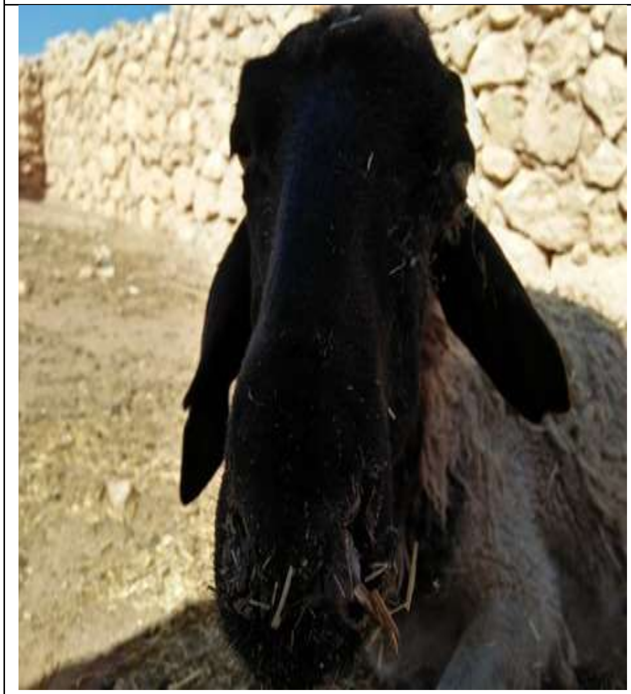
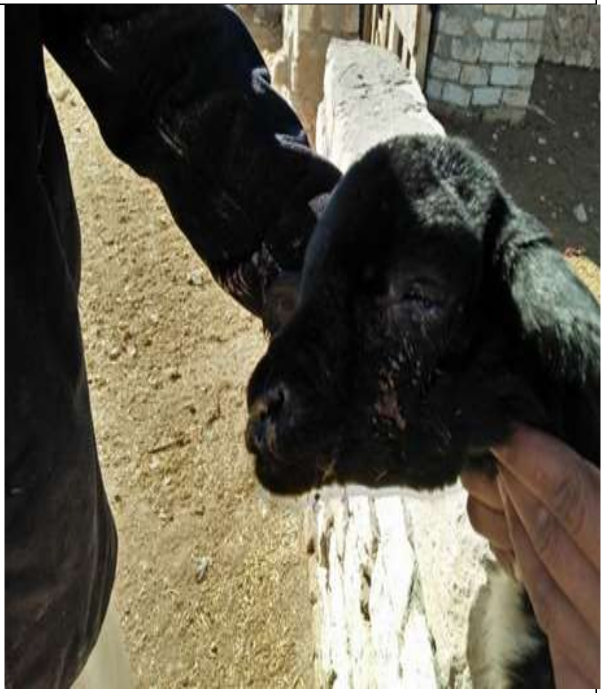

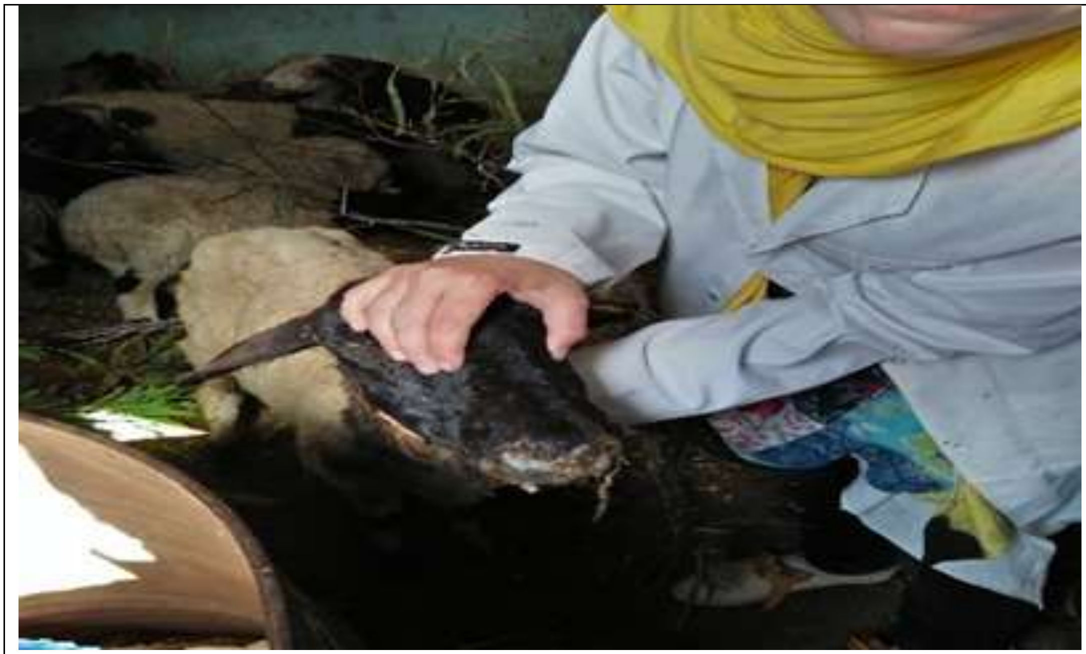

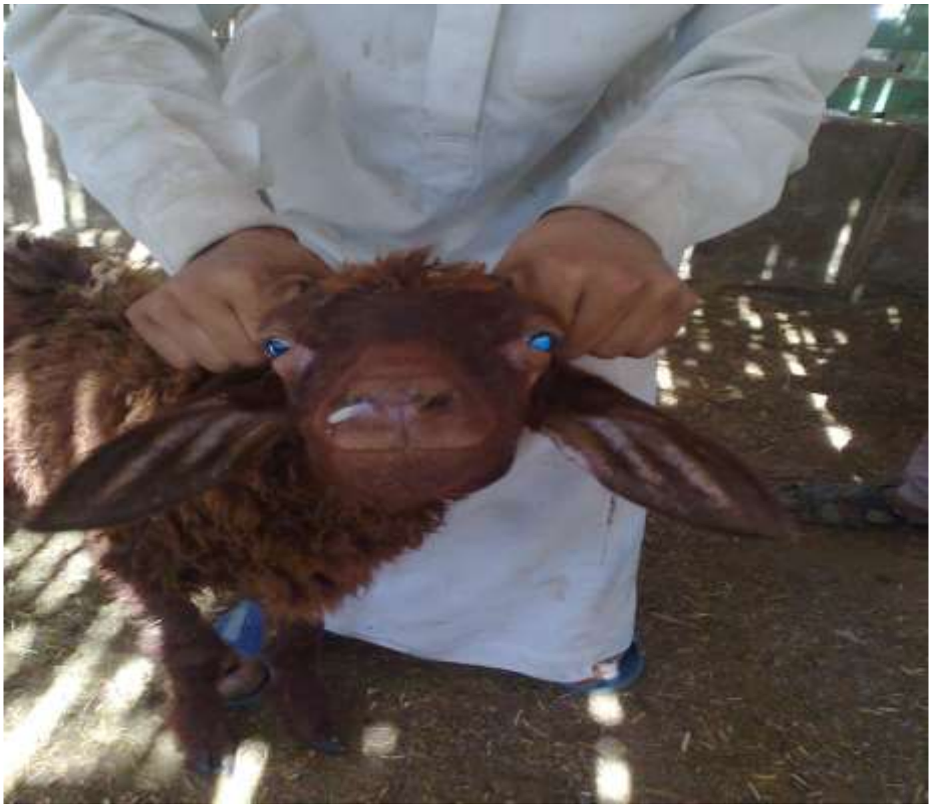

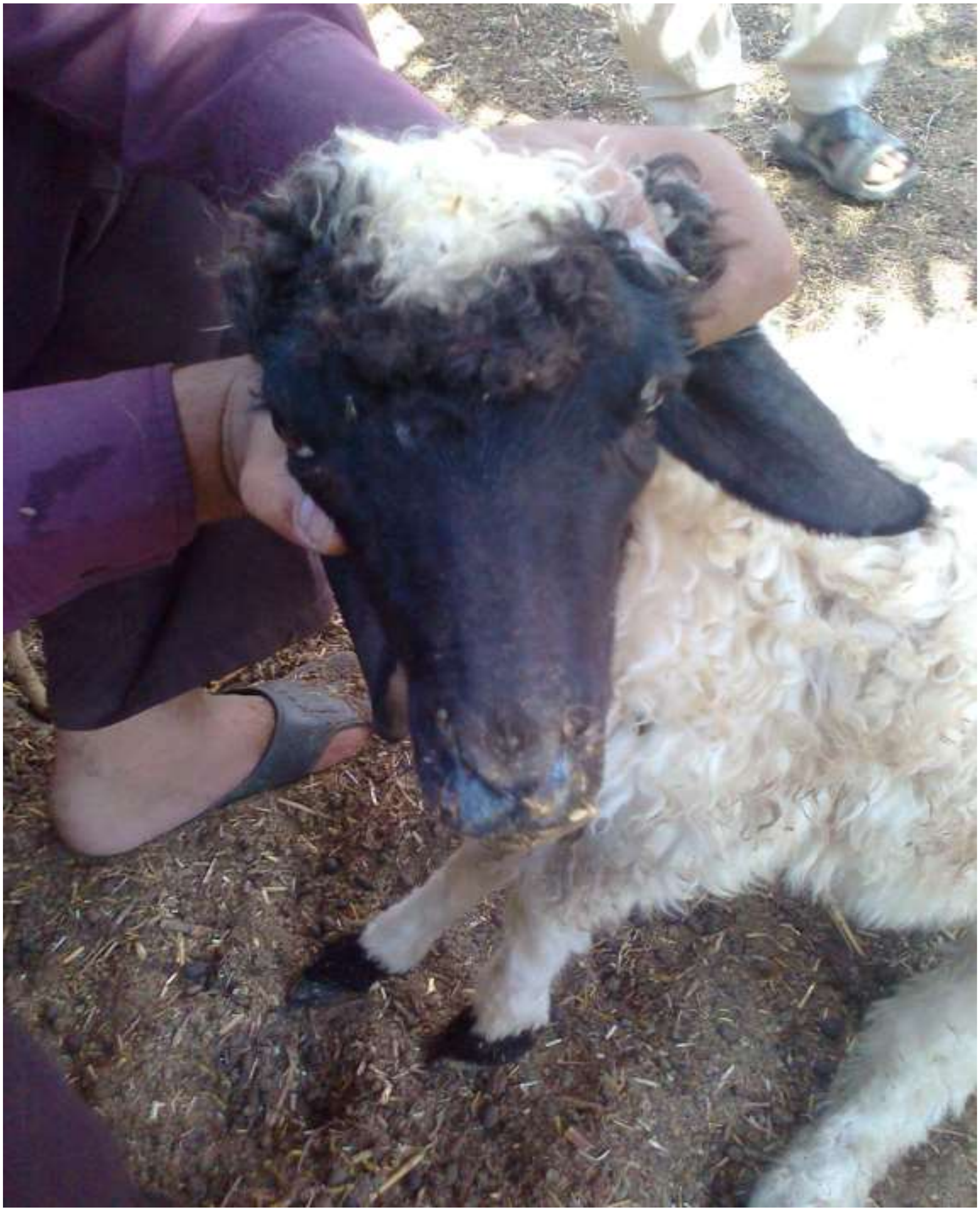

Figure S1: Sheep showing signs of pneumonia

```
NM_001048231.1      GCCTGGCTCCAGGCCAAGAGGAAGCCCAGGAAGGCTCCCCGCAGGGACCTCTGCTACGAC    60
H                   GCCTGGCTCCAGGCCAAGAGGAAGCCCAGGAAGGCTCCCCGCAGGGACCTCTGCTACGAC    60
P                   GCCTGGCTCCAGGCCAAGAGGAAGCCCAGGAAGGCTCCCCGCAGGGACCTCTGCTACGAC    60
                    ************************************************************

NM_001048231.1      GCCTTTGTGTCCTACAGCGAGCGGGATTCCTACTGGGTGGAGAACCTCATGGTCCAGGAG    120
H                   GCCTTTGTGTCCTACAGCGAGCGGGATTCCTACTGGGTGGAGAACCTCATGGTCCAGGAG    120
P                   GCCTTTGTGTCCTACAGCGAGCGGGATTCCTACTGGGTGGAGAACCTCATGGTCCAGGAG    120
                    ************************************************************

NM_001048231.1      CTGGAGCACTTCAACCCTCCCTTTAAGCTGTGTCTTCATAAGCGAGACCTTGTCCCTGGC    180
H                   CTGGAGCACTTCAACCCTCCCTTTAAGCTGTGTCTTCATAAGCGAGACCTTGTCCCTGGC    180
P                   CTGGAGCACTTCAACCCTCCCTTTAAGCTGTGTCTTCATAAGCGAGACTTTGTCCCTGGC    180
                    ************************************************ ***********

NM_001048231.1      AAATGGATTATCGACAACATCATCGACTCCATTGAAAAGAGCCGCAAAACCATCTTTGTG    240
H                   AAATGGATTATCGACAACATCATCGACTCCATTGAAAAGAGCCGCAAAACCATCTTTGTG    240
P                   AAATGGATTATCGACAACATCATCGACTCCATTGAAAAGAGCCGCAAAACCATCTTTGTG    240
                    ************************************************************

NM_001048231.1      CTTTCGGAGAGCTTTGTGAGGAGCGAGTGGTGCAAGTATGAGCTGGACTTCTCCCACTTC    300
H                   CTTTCGGAGAGCTTTGTGAGGAGCGAGTGGTGCAAGTATGAGCTGGACTTCTCCCACTTC    300
P                   CTTTCGGAGAGCTTTGTGAGGAGCGAGTGGTGCAAGTATGAGCTGGACTTCTCCCACTTC    300
                    ************************************************************

NM_001048231.1      CGTCTCTTTGATGAGAACAACGATGCTGCCATTCTGATTCTGCTGGAGCCCATT354
H                   CGTCTCTTTGATGAGAACAACGATGCTGCCATTCTGATTCTGCTGGAGCCCATT354
P                   CGTCTCTTTGATGAGAACAACGATGCTGCCATTCTGATTCTGCTGGAGCCCATT354
                    ******************************************************
```

Figure S2: Representative alignment of the TLR2 gene (354 bp) between healthy (H) and pneumonia affected ewes (P) compared to reference GenBank accession number (NM_001048231.1).

```
XM_042247586.1      ATTCATCCACATCACCAGCATCACAAAGCACAGAGAGAAGATGCTTTTCTTCCCAGCTAT   60
H                   ATTCATCCACATCACCAGCATCACAAAGCACAGAGAGAAGATGCTTTTCTTCCCAGCTAT   60
P                   ATTCATCCACATCACCAGCATCACAAAGCACAGAGAGAAGATACTTTTCTTCCCAGCTAT   60
                    ****************************************** *****************

XM_042247586.1      TCTCATGGGCCATTGCTGGCACCTCCATCCTGCTCCTGAGTGCCTGTTTTATCACCAGAT   120
H                   TCTCATGGGCCATTGCTGGCACCTCCATCCTGCTCCTGAGTGCCTGTTTTATCACCAGAT   120
P                   TCTCATGGGCCATTGCTGGCACCTCCATCCTGCTCCTGAGTGCCTGTTTTATCACCAGAT   120
                    ************************************************************

XM_042247586.1      GTGTTGTGACATATGGCATCTTTCAACTATGTGATGAGAAAAAGTTCCAGCCACCTGGGG   180
H                   GTGTTGTGACATATGGCATCTTTCAACTATGTGATGAGAAAAAGTTCCAGCCACCTGGGG   180
P                   GTGTTGTGACATATGGCATCTTTCAACTATGTGATGAGAAAAAGTTCCAGCCACCTGGGG   180
                    ************************************************************

XM_042247586.1      ATTCCATGGAACTCTCCTGCTATAATGATGGATCAGGTTCAGTCAAGAATTGCTGTCCAC   240
H                   ATTCCATGGAACTCTCCTGCTATAATGATGGATCAGGTTCAGTCAAGAATTGCTGTCCAC   240
P                   ATTCCATGGAACTCTCCTGCTATAATGATGGATCAGGTTCAGTCAAGAATTGCTGTCCAC   240
                    ************************************************************

XM_042247586.1      TGAACTGGGTCCATTTTCAATCCAGCTGCTACTTCTTTTCTGCTGACACCATGTCCTGGG   300
H                   TGAACTGGGTCCATTTTCAATCCAGCTGCTACTTCTTTTCTGCTGACACCATGTCCTGGG   300
P                   TGAACTGGGTCCATTTTCAATCCAGCTGCTACTTCTTTTCTGCTGACACCATGTCCTGGG   300
                    ************************************************************

XM_042247586.1      CAGCAAGTCTAAAAAACTGCTCAAGCATGGGCGCTCACCTGGTGGTTATCAACACGCAGG   360
H                   CAGCAAGTCTAAAAAACTGCTCAAGCATGGGCGCTCACCTGGTGGTTATCAACACGCAGG   360
P                   CAGCAAGTCTAAAAAACTGCTCAAGCATGGGCGCTCACCTGGTGGTTATCAACACGCAGG   360
                    ************************************************************

XM_042247586.1      AGGAGCAGGAATTCCTTTACCATGCAAAACCTAGAAAGAAAGAGTTTTATATTGGACTGA   420
H                   AGGAGCAGGAATTCCTTTACCATGCAAAACCTAGAAAGAAAGAGTTTTATATTGGACTGA   420
P                   AGGAGCAGGAATTCCTTTACCATGCAAAACCTAGAAAGAAAGAGTTTTATATTGGACTGA   420
                    ************************************************************

XM_042247586.1      CGGACCAGGTGGTCGAGGGTCAC   443
H                   CGGACCAGGTGGTCGAGGGTCAC   443
P                   CGGACCAGGTGGTCGAGGGTCAC   443
                    ***********************
```

Figure S3: Representative alignment of the CLEC4E gene (443 bp) between healthy (H) and pneumonia affected ewes (P) compared to reference GenBank accession number (XM_042247586.1).

```
XM_004003220.5      CTGGAGGAGCTGCGGCGGACGCGGGCCGACCTCCGAGCTGTGCAGGGCTGGGCAGTCAGC   60
H                   CTGGAGGAGCTGCGGCGGACGCGGGCCGACCTCCGAGCTGTGCAGGGCTGGGCAGTCAGC   60
P                   CTGGAGGAGCTGCGGCGGACGCGGGCCGACCTCCGAGCTGTGCAGGGCTGGGCAGTCAGC   60
                    ************************************************************

XM_004003220.5      CGCTGGCTGCCGGCAGGTTGTGAAACAGCGATTTTATTCCCCATGCGTTCCAAGAAGATT   120
H                   CGCTGGCTGCCGGCAGGTTGTGAAACAGCGATTTTATTCCCCATGCGTTCCAAGAAGATT   120
P                   CGCTGGCTGCCGGCAGGTTGTGAAACAGCCATTTTATTCCCCATGCGTTCCAAGAAGATT   120
                    ***************************** ******************************

XM_004003220.5      TTTGCAAGCGTGCATCCGGTGACACCAATGAAACTTGAGAGTTTCAGTGCCTGCATTTGG   180
H                   TTTGCAAGCGTGCATCCGGTGACACCAATGAAACTTGAGAGTTTCAGTGCCTGCATTTGG   180
P                   TTTGCAAGCGTGCATCCGGTGACACCAATGAAACTTGAGAGTTTCAGTGCCTGCATTTGG   180
                    ************************************************************

XM_004003220.5      GTCAAAGCCACAGAAGTATTAAACAAAACAGTCCTGTTTTCCTATGGCACAAAAAGGAAT   240
H                   GTCAAAGCCACAGAAGTATTAAACAAAACAGTCCTGTTTTCCTATGGCACAAAAAGGAAT   240
P                   GTCAAAGCCACAGAAGTATTAAACAAAACAGTCCTGTTTTCCTATGGCACAAAAAGGAAT   240
                    ************************************************************

XM_004003220.5      CCATATGAGATCCAGCTGTACCTCAGCTATCGGTCCATAATGCTTGTGGTGGGTGGAGAG   300
H                   CCATATGAGATCCAGCTGTACCTCAGCTATCGGTCCATAATGTTTGTGGTGGGTGGAGAG   300
P                   CCATATGAGATCCAGCTGTACCTCAGCTATCGGTCCATAATGCTTGTGGTGGGTGGAGAG   300
                    ****************************************** *****************

XM_004003220.5      GAAAACAGACTGGTCGCTGATGCTGTGATTTCCCCAGGAACATGGACCCATCTGTGCAGC   360
H                   GAAAACAGACTGGTCGCTGATGCTGTGATTTCCCCAGGAACATGGACCCATCTGTGCAGC   360
P                   GAAAACAGACTGGTCGCTGATGCTGTGATTTCCCCAGGAACATGGACCCATCTGTGCAGC   360
                    ************************************************************

XM_004003220.5      ACC     363
H                   ACC     363
P                   ACC     363
                    ***
```

Figure S4: Representative alignment of the PTX3 gene (363 bp) between healthy (H) and pneumonia affected ewes (P) compared to reference GenBank accession number (XM_004003220.5).

```
XM_027974200.3      ACCTTCCTCATCCGCGACAGCTCGGACCAGCGCCACTTCTTCACCCTCAGCGTCAAGACC   60
H                   ACCTTCCTCATCCGCGACAGCTCGGACCAGCGCCACTTCTTCACCCTCAGCGTCAAGACC   60
P                   ACCTTCCTCATCCGCGACAGCTCGGACCAGCGCCACTTCTTCACCCTCAGCGTCAAGACC   60
                    ************************************************************

XM_027974200.3      CAGTCGGGGACCAAGAACCTGCGCATCCAGTGCGAGGGGGGCAGCTTCTCTCTGCAGAGC   120
H                   CAGTCGGGGACCAAGAACCTGCGCATCCAGTGCGAGGGGGGCAGCTTCTCTCTGCAGAGC   120
P                   CAGTCGGGGACCAAGAACCTGCGCATCCAGTGCGAGGGGGGCAGCTTCTCTCTGCAGAGC   120
                    ************************************************************

XM_027974200.3      GACCCCCGGAGCACGCAGCCCGTGCCCCGCTTCGACTGCGTGCTCAAGCTGGTGCATCAC   180
H                   GACCCCCGGAGCACGCAGCCCGTGCCCCGCTTCGACTGCGTGCTCAAGCTGGTGCATCAC   180
P                   GACCCCCGGAGCACGCAGCCCGTGCCCCGCTTCGACTGCGTGCTCAAGCTGGTGCATCAC   180
                    ************************************************************

XM_027974200.3      TACATGCCCGCCGCCGGCGCCCCCTCGTTCTCCCCGCCCCCCGCTGAACCCTCCTCCTCG   240
H                   TACATGCCCGCCGCCGGCGCCCCCTCGTTCTCCCCGCCCCCCGCTGAACCCTCCTCCTCG   240
P                   TACATGCCCGCCGCCGGCGCCCCCTCGTTCTCCCCGCCCCCCGCTGAACCCTCCTCCTCG   240
                    ************************************************************

XM_027974200.3      CCCTCCTCCGAGGTGCCCGAGCAGCCACCGGCCCAGCCGCTCCCCGGGAGCCCCCCCAGG   300
H                   CCCTCCTCCGAGGTGCCCGAGCAGCCACCGGCCCAGCCGCTCCCCGGGAGCCCCCCCAGG   300
P                   CCCTCCTCCGAGGTGCCCGAGCAGCCACCGGCCCAGCCGCTCCCCGGGAGCCCCCCCAGG   300
                    ************************************************************

XM_027974200.3      AGAGCCTATTACATTTACTCGGGGGGCGAGAAGATCCCTCTGGTGTTGAGCCGGCCCCTC   360
H                   AGAGCCTATTACATTTACTCGGGGGGCGAGAAGATCCCTCTGGTGTTGAGCCGGCCCCTC   360
P                   AGAGCCTATTACATTTACTCGGGGGGCGAGAAGATCCCTCTGGTGTTGAGCCGGCCCCTC   360
                    ************************************************************

XM_027974200.3      TCCTCCAACGTGGCCACTCTCCAACATCTCTGTCGGAAGACCGTCAACGGCCACCTGGAC   420
H                   TCCTCCAACGTGGCCACTCTCCAACATCTCTGTCGGAAGACCGTCAACGGCCACCTGGAC   420
P                   TCCTCCAACGTGGCCACTCTCCAACATCTCTGTCGGAAGACCGTCAACGGCCACCTGGAC   420
                    ************************************************************

XM_027974200.3      TCCTACGAGAAAGTCACGCAGCTGCCTGGGCCCATTCGGGAGTTCCTGGACCAGTACGAT   480
H                   TCCTACGAGAAAGTCACGCAGCTGCCTGGGCCCATTCGGGAGTTCCTGGACCAGTACGAT   480
P                   TCCTACGAGAAAGTCACCCAGCTGCCTGGGCCCATTCGGGAGTTCCTGGACCAGTACGAT   480
                    ***************** ******************************************
```

Figure S5: Representative alignment of the SOCS3 gene (480 bp) between healthy (H) and pneumonia affected ewes (P) compared to reference GenBank accession number (XM_027974200.3).

```
NM_001009401.2      ATGACTTCCAAGCTGGCTGTTGCTCTCTTGGCCGCTTTCCTGCTCTCTGCAGCTCTGTGT   60
H                   ATGACTTCCAAGCTGGCTGTTGCTCTCTTGGCCGCTTTCCTGCTCTCTGCAGCTCTGTGT   60
P                   ATGACTTCCAAGCTGGCTGTTGCTCTCTTGGCCGCTTTCCTGCTCTCTGCAGCTCTGTGT   60
                    ************************************************************

NM_001009401.2      GAAGCTGCAGTTCTGTCAAGAATGAGTACAGAACTTCGATGCCAATGCATAAAAACACAT   120
H                   GAAGCTGCAGTTCTGTCAAGAATGAGTACAGAACTTCGATGCCAATGCATAAAAACACAT   120
P                   GAAGCTGCAGTTCTGTCAAGAATGAGTACAGAACTTCGATGCCAATGCATAAAAACACAT   120
                    ************************************************************

NM_001009401.2      TCCACACCTTTCCACCCCAAATTTATCAAAGAACTGAGAGTTATTGAGAGTGGGCCACAC   180
H                   TCCACACCTTTCCACCCCAAATTTATCAAAGAACTGAGAGTTATTGAGAGTGGGCCACAC   180
P                   TCCACACCTTTCCACCCCAAATTTATCAAAGAATTGAGAGTTATTGAGAGTGGGCCACAC   180
                    ********************************* **************************

NM_001009401.2      TGCGAAAATTCAGAAATCATTGTTAAGCTTACCAACGGAAAAGAGGTGTGCTTAGACCCC   240
H                   TGCGAAAATTCAGAAATCATTGTTAAGCTTACCAACGGAAAAGAGGTGTGCTTAGACCCC   240
P                   TGCGAAAATTCAGAAATCATTGTTAAGCTTACCAACGGAAAAGAGGTGTGCTTAGACCCC   240
                    ************************************************************

NM_001009401.2      AAGGAAAAGTGGGTGCAGAAGGTTGTGCAGGCATTTTTGAAGAGAGCTGAGAAGCAAGAT   300
H                   AAGGAAAAGTGGGTGCAGAAGGTTGTGCAGGCATTTTTGAAGAGAGCTGAGAAGCAAGAT   300
P                   AAGGAAAAGTGGGTGCAGAAGGTTGTGCAGGCATTTTTGAAGAGAGCTGAGAAGCAAGAT   300
                    ************************************************************
```

Figure S6: Representative alignment of the CXCL8 gene (300 bp) between healthy (H) and pneumonia affected ewes (P) compared to reference GenBank accession number (NM_001009401.2).

```
XM_042230436.2      CCGGCCACGCCGGGCATCACCTGCCCGACTCCCACATCCGTGGAGCATGCAGACATCCAG    60
H                   CCGGCCACGCCGGGCATCACCTGCCCGACTCCCACATCCGTGGAGCATGCAGACATCCAG    60
P                   CCGGCCACGCCGGGCATCACCTGCCCGACTCCCACATCCGTGGAGCATGCAGACATCCAG    60
                    ************************************************************

XM_042230436.2      GTCAAGAATTACAGCATCAACTCCAGGGAGCGGTATGTTTGTAATTCTGGCTTCAAGCGT    120
H                   GTCAAGAATTACAACATCAACTCCAGGGAGCGGTATGTTTGTAATTCTGGCTTCAAGCGT    120
P                   GTCAAGAATTACAGCATCAACTCCAGGGAGCGGTATGTTTGTAATTCTGGCTTCAAGCGT    120
                    ************* **********************************************

XM_042230436.2      AAAGCTGGGACCTCCAGCTTGACCCAGTGTGTGTTTAACGAGACCGCGAAAGTCGCCCAC    180
H                   AAAGCTGGGACCTCCAGCTTGACCCAGTGTGTGTTTAACGAGACCGCGAAAGTCGCCCAC    180
P                   AAAGCTGGGACCTCCAGCTTGACCCAGTGTGTGTTTAACGAGACCGCGAAAGTCGCCCAC    180
                    ************************************************************

XM_042230436.2      TGGACCACTCCCAACCTCAAGTGCATCAGAGACCCCTCCCTGAGTCACCAAAGGCCACCC    240
H                   TGGACCACTCCCAACCTCAAGTGCATCAGAGACCCCTCCCTGAGTCACCAAAGGCCACCC    240
P                   TGGACCACTCCCAACCTCAAGTGCATCAGAGACCCCTCCCTGAGTCACCAAAGGCCACCC    240
                    ************************************************************

XM_042230436.2      TCCACAGCAGCGCCGACAGGGTTGACCCCAGAGCCAGAGAGCCCCACCCCCTCCGGAAAA    300
H                   TCCACAGCAGCGCCGACAGGGTTGACCCCAGAGCCAGAGAGCCCCACCCCCTCCGGAAAA    300
P                   TCCACAGCAGCGCCGACAGGGTTGACCCCAGAGCCAGAGAGCCCCACCCCCTCCGGAAAA    300
                    ************************************************************

XM_042230436.2      GGTGCATATCAGCACAATCCCAGCGTTGTGACCGCTGTCTCCATACCTGTCGCTGTTGTC    360
H                   GGTGCATATCAGCACAATCCCAGCGTTGTGACCGCTGTCTCCATACCTGTCGCTGTTGTC    360
P                   GGTGCATATCAGCACAATCCCAGCGTTGTGACCGCTGTCTCCATACCTGTCGCTGTTGTC    360
                    ************************************************************

XM_042230436.2      TTTGCAGTGTGCCTGGTG          378
H                   TTTGCAGTGTGCCTGGTG          378
P                   TTTGCAGTGTGCCTGGTG          378
                    ******************
```

Figure S7: Representative alignment of the IL15RA gene (378 bp) between healthy (H) and pneumonia affected ewes (P) compared to reference GenBank accession number (XM_042230436.2).
